# The Medical Student Performance Evaluation in global medical education: addressing a missing Middle Eastern perspective

**DOI:** 10.3389/fmed.2026.1786429

**Published:** 2026-03-16

**Authors:** Abdullah Jabri, Bader Taftafa, Mohamed Alsharif, Abdulaziz Mhannayeh, Dania Sibai, Abderrahman Ouban, Fouad F. Jabri

**Affiliations:** 1 College of Medicine, Alfaisal University, Riyadh, Saudi Arabia; 2 Department of Global Public Health, Karolinska Institutet, Stockholm, Sweden

**Keywords:** faculty development, global medical education, learner assessment, Medical Student Performance Evaluation (MSPE), residency selection, undergraduate medical education

## Abstract

**Background:**

An essential part of the residency selection process is the Medical Student Performance Evaluation (MSPE), which provides an organized overview of the performance of undergraduate medical students. Despite the Association of American Medical Colleges’ (AAMC) extensive guidelines, there is still considerable variation in MSPE content, interpretation, and application among schools. Furthermore, limited published data exist regarding the adaptation and application of MSPE frameworks outside of North America.

**Methods:**

This narrative review was conducted using a structured literature search of PubMed/MEDLINE and Google Scholar to identify peer-reviewed articles, national guidelines, and policy documents addressing MSPE structure, implementation, standardization, and program director perspectives. Relevant literature was synthesized thematically. In addition, the establishment and implementation of the MSPE at Alfaisal University were examined as an institutional case example.

**Results:**

The review summarizes the MSPE’s historical evolution and examines ongoing challenges related to grading variability, narrative evaluation, professionalism reporting, transparency, comparability, and standardization. It also explores the presence of MSPE-equivalent systems in the Middle East, where peer-reviewed analyses and formalized guidance remain limited. The Alfaisal University model demonstrates how systematic data collection, narrative clarity, and structured faculty participation can support the implementation of standardized evaluation frameworks in a non-North American educational setting.

**Conclusion:**

By situating an institutional experience within the broader medical education literature, this review contributes to discussions on learner evaluation, cross-institutional comparability, and the globalization of assessment standards. The observations presented may inform medical schools, educators, and policymakers seeking to develop fair and transparent performance evaluation systems across diverse educational contexts.

## 1 Introduction

### 1.1 Purpose and role in residency selection

The Medical Student Performance Evaluation (MSPE) is an institutional, standardized evaluation that synthesizes a student’s experiences, attributes, and academic performance for residency programs. It should, whenever feasible, contextualize performance in relation to institutional peers and be an objective assessment rather than a recommendation (1, 2). In contemporary selection, the MSPE remains widely used: in the 2024 NRMP Program Director Survey, 85% of program directors reported using the MSPE/Dean’s Letter when selecting applicants to interview (mean importance 4.1/5) (3).

### 1.2 Residency selection before the MSPE

Before formal MSPE standardization, programs relied on heterogeneous “Dean’s Letters,” basic-science and clerkship grades, licensing-exam scores, and ad-hoc recommendation letters. Early work and AAMC guidance identified persistent problems: large variability in format/content and limited comparative data, which hindered cross-applicant evaluation (1, 4). As seen in Table 1, these inputs lacked standardization and cross-institutional comparability.

**Table 1:** Pre-MSPE vs. MSPE era: inputs and implications in residency selection (1, 2).

Era	Primary evaluative documents emphasized in residency applications	Comparative data available?	Known issues	Implications for selection
Pre-MSPE (pre-2002)	Dean’s Letter (heterogeneous), basic-science and clerkship grades, USMLE scores, individual LORs	Often limited/absent	Format variability; scarce peer comparisons; emphasis on preclinical signals	Difficult cross-school comparisons; heavier reliance on exams
MSPE era (2002–2016)	Structured MSPE; narratives across core clerkships; professionalism; appendices with graphs (varied)	Improving but inconsistent	Uneven adoption; unclear adjective legends; variable data displays	Better synthesis; still variability for PDs
MSPE (2016/2017 → present)	Revised template: ACGME competencies foregrounded; NCs; explicit grade derivations; co-located comparative graphics	Encouraged; quality varies by school	Narrative vagueness; grade inflation; inconsistent adherence	More holistic read; value depends on transparency & comparability

This table contrasts the residency selection elements used before and after the MSPE framework was established, highlighting the variability in formats and comparative data availability.

USMLE, United States medical licensing examination; LORs, letters of recommendation; MSPE, Medical Student Performance Evaluation; PDs, program directors.

### 1.3 Origins and evolution of the MSPE

#### 1.3.1 1989–2001: seeds of standardization

In 1989, an AAMC committee issued national guidance for Dean’s Letters, emphasizing evaluative summaries and comparative context. Limited adoption and uneven comparative reporting prompted a second advisory effort (1, 4).

#### 1.3.2 2002: from “Dean’s Letter” to MSPE

In 2002, the document was renamed the Medical Student Performance Evaluation to reaffirm its evaluative purpose and prescribe a structured format spanning academic history, preclinical and clinical performance, professionalism, and a comparative summary, with appendices supplying peer-comparison graphics (1).

#### 1.3.3 2014–2017: modernization and deeper standardization

An AAMC MSPE Task Force (2014–2017) recommended a revised template highlighting ACGME competencies; relocating comparative graphics into the body of the letter; renaming “Unique Characteristics” to “Noteworthy Characteristics” (limited to three concise bullets); clarifying grading derivations and weightings; and capping length. The guidelines urged schools to reveal the extent to which clinical performance influences grades and specifically stated that clerkship evaluations are frequently the most important section for interview selection and ranking (2, 5).

#### 1.3.4 From 2016 until now: implementation of the modern MSPE framework

The need for transparent comparative data is reinforced by studies that show variability in adherence and interpretability despite improvements (e.g., inconsistent use of final adjectives and limited comparative professionalism data) (6–8).

### 1.4 Shift toward clinical performance–based evaluation

Previous materials, especially those published before 2002, frequently emphasized course/basic-science results without providing consistent, comparable summaries of clinical performance or professionalism. The 2002 MSPE shifted the evaluative center toward *core clerkship narratives* and professionalism, supported by peer-comparison graphics (1). The 2016/2017 revisions went further by requiring disclosure of clerkship grade components and weighting (e.g., clinical evaluations vs. shelf/OSCE), thereby allowing readers to distinguish bedside ability from test performance and reflecting program directors’ emphasis on clinical narratives (2, 5). Standardized licensing examinations such as USMLE remained part of residency applications across all eras; however, the MSPE reforms shifted the evaluative emphasis toward structured clinical performance and comparative institutional reporting rather than exam-centric signals.

### 1.5 Practical significance and timelines

Standardization improves cross-applicant comparability while enabling holistic review by synthesizing academic, professional, and contextual information. Procedurally, ERAS timelines align MSPE availability with early residency application review cycles (9, 10).

## 2 Structure and standardization of the MSPE

### 2.1 Core components of the MSPE

The Medical Student Performance Evaluation (MSPE) includes several core sections that program directors use to evaluate applicants, though the perceived usefulness of these sections differs across schools. The Noteworthy Characteristics (NC) section is designed to highlight distinctive aspects of a student’s background or achievements. However, program directors frequently regard it as one of the least useful components, but students perceive self-authorship of NCs as a potential advantage in the match process (6, 11, 12). In contrast, the academic history summary is often regarded as one of the most objective and dependable predictors of future residency performance (11, 13).

Clerkship evaluations and grades, particularly those from core rotations such as internal medicine, play a significant role in residency selection. Yet, their value is undermined by variation in grading systems, incomplete reporting, and ongoing concerns about grade inflation (6, 14–16). The summary paragraph and final adjective (e.g., “outstanding,” “excellent,” “very good”) often weigh heavily in shaping impressions, at times more than the narrative descriptions themselves, though their use differs considerably between institutions (8, 16, 17).

Narrative evaluations aim to provide a richer picture of student performance, but they are frequently weakened by vague wording, coded language, and evaluator bias. These shortcomings have led to initiatives focused on faculty development to improve clarity and fairness (11, 18). Comparative or graphic data displays, when present, are considered especially valuable by program directors, but many schools either omit them or fail to follow AAMC recommendations for transparency (13–15, 19).

Finally, professionalism tests are regularly recognized as crucial in residency selection, despite concerns about their uniformity and standardization (6, 11). Overall, while the MSPE covers significant areas, recurrent issues of inconsistency, lack of openness, and poor adherence to AAMC principles restrict its effectiveness as a genuinely standardized evaluation tool.

### 2.2 How MSPEs are written

The process of drafting an MSPE differs widely between institutions, reflecting variations in authorship, data collection, and stakeholder involvement. In many cases, faculty or Student Affairs offices write the document, but concerns about conflicts of interest, limited transparency, and uneven accountability remain (8, 20). Some schools allow students to draft their own NCs, a practice that can increase engagement but raises concerns about fairness compared to schools that prohibit self-authorship (6, 12).

Data collection also shows inconsistencies, particularly in grading practices. Differences in clerkship grading systems, terminology, and distributions, together with evidence of grade inflation, undermine reliability (15, 16). Narrative evaluations, which serve as the backbone of clerkship assessments, are particularly susceptible to bias and overly polite or coded language. As a result, faculty training and structured feedback processes have been introduced to improve their quality (11, 18).

Stakeholder engagement has been identified as an important area for improvement. Experts have advocated for greater collaboration between undergraduate and graduate medical education, as well as national organizations, to improve MSPE standards, increase transparency, and better align evaluations with competency-based milestones (9, 11). These dynamics show that the MSPE is more than just a paper; it reflects broader institutional culture and systemic behaviors that have a direct impact on residency results.

### 2.3 Standardization efforts and variation

The Association of American Medical Colleges (AAMC) attempted to standardize the MSPE by first establishing standards in 2002 and then modifying them in 2016. Despite these efforts, implementation is inconsistent across schools. The inclusion of a comparative summative term was intended to promote transparency and impartiality, but research indicates that it frequently carries more weight than narrative evaluations, creating questions about balance (7).

Directors of residency programs express varying degrees of confidence in the MSPE; many are dubious of its validity and demand more class rank and comparison statistics, particularly in light of the USMLE Step 1 switch to pass/fail (8). Only a small percentage of schools provide thorough descriptors and grade distributions, and grade inflation is still on the rise, according to retrospective studies of MSPEs, which reveal significant variation in adherence to AAMC requirements (10). Differences in the way schools report clerkship assessments, the use of words rather than numerical descriptors, and the inclusion of preclinical or fourth-year performance are all confirmed by large-scale analyses (12).

Although the 2016 Task Force addressed several shortcomings, many believe the MSPE still lacks openness and uniformity. Stronger coordination across institutions and organizations is required to strengthen accountability and link the MSPE more closely with competency-based frameworks (18, 20). Overall, these data illustrate that, despite efforts to standardize the MSPE, it remains highly variable, limiting its fairness and utility in residency selection.

### 2.4 Specialty-specific adaptation

While the MSPE is not expressly altered for specific specialties, the perceived value differs by discipline. A survey of program directors from 28 disciplines indicated variations in how the MSPE is used. Approximately one-third of directors deemed it highly influential in screening, with professionalism continuously recognized as the most important component, and NCs were almost uniformly regarded as the least valuable (6).

Specialty-based perspectives also appear in studies of student-authored NCs. For instance, emergency medicine applicants reported finding the section least useful, while psychiatry applicants were most dissatisfied with the strict word limits (12). Similarly, the weight given to core clerkships differs by specialty. Internal medicine clerkship performance has the greatest influence on residency decisions, while neurology and psychiatry performance is frequently regarded as less important (6).

These findings indicate that, while MSPEs are not directly suited to certain disciplines, their interpretation and impact differ by expertise, making them context-dependent in practice.

## 3 The development and implementation of the MSPE at Alfaisal university

At Alfaisal University, the MSPE has been developed in alignment with principles articulated in the AAMC MSPE Task Force recommendations (2017) and related standardization guidance including structured narrative synthesis, transparent comparative data, defined professionalism reporting, and disclosure of grading derivations (2, 21).

### 3.1 Overview and local context

**Figure 1:**
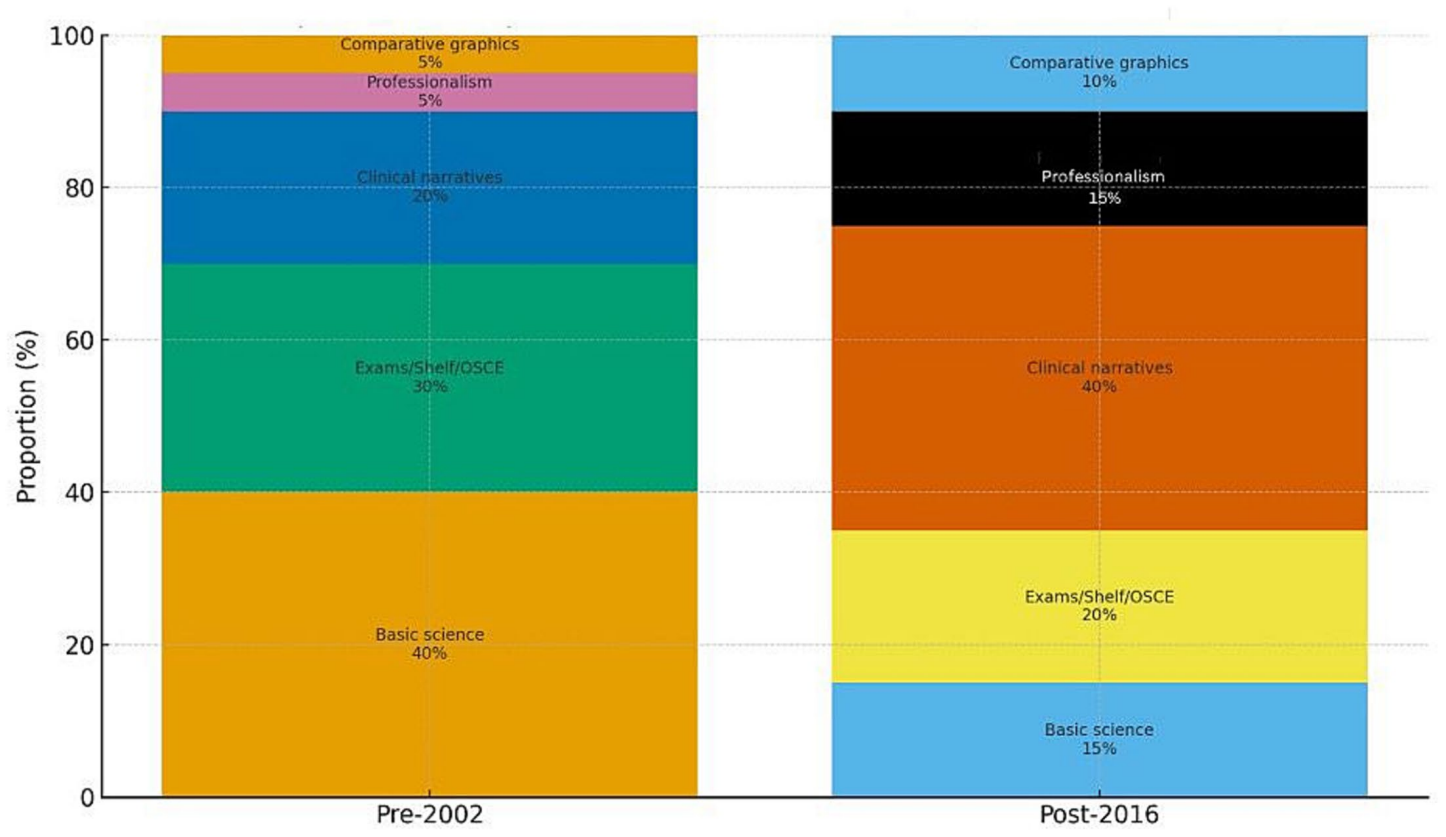
Illustrative shift from basic-science signals to clinical performance emphasis in the MSPE evaluation, showing changes pre- and post-2016 revisions.

Alfaisal University implemented the MSPE framework in 2016, and it has since been an integral part of how the institution presents student performance to residency programs. The university strictly adheres to the AAMC MSPE Task Force structure and expectations, with a focus on transparency, curricular context, and narrative clarity (21). Because the MSPE at Alfaisal is designed for students applying both abroad and inside the region, the institution has continuously developed its approach to meet the needs of various residencyms paths. This alignment with internationally recognized guidelines has informed the structure and presentation of the MSPE, enhancing its clarity for external reviewers. The Alfaisal MSPE has consistently incorporated core components recommended by existing MSPE guidance as GPA, academic distinctions, and clerkship narratives, as shown in Table 2. This table highlights Alfaisal’s approach to selecting which elements are essential for inclusion in the MSPE and the criteria for conditional inclusion (see Figure 1).

**Table 2:** Elements included or excluded from the Alfaisal MSPE (policy overview).

Element	Included?	Source/owner	How it appears in the MSPE	Policy notes
GPA/academic distinctions	Yes	Assessment Office	Academic History; concordant with transcript	Reported as per policy; cross-checked with Registrar
USMLE scores	Conditional	Student-provided/Registrar (if stored)	Included only if institutional policy permits	Respect privacy and ERAS guidance
Clerkship narratives	Yes	Clinical departments	Integrated narrative summaries for core rotations	Faculty development to reduce vague language
Clerkship grade distributions	Yes	Assessment Office	Comparative graphics or percentile position	Encouraged for transparency; legend included
NBME shelf/OSCE	Conditional/yes	Assessment Office	Percentiles or cohort comparison (if allowed)	Follow local policy on exam reporting
Professionalism/remediation	Yes	UME Leadership/Student Affairs	Standardized descriptors; outcomes if applicable	Use institutional glossary; protect confidentiality
Noteworthy Characteristics (NC)	Yes	Student interview + MSPE team	Three concise, vetted statements	One-to-one interview ensures accuracy/professionalism
Extracurricular/leadership	Conditional	Student Affairs (verified)	Included when substantively relevant to professional development	Avoid padding; require verification
Research/publications	Conditional	Student Affairs (verified)	Summarized when impactful; detailed list in CV, not MSPE	Ensure verifiability and relevance
Specialty-specific tailoring	Yes	MSPE team	Highlights achievements, experiences, and skills most relevant to the student’s intended specialty (e.g., internal medicine, general surgery)	Ensures content remains factual and evidence-based while emphasizing strengths that support the student’s specialty interest

This table summarizes the key components considered in the Alfaisal MSPE, including which elements are consistently included (e.g., GPA, clerkship narratives) and which are conditionally included based on relevance (e.g., extracurriculars, research contributions). GPA, Grade Point Average; NBME, National Board of Medical Examiners; OSCE, Objective Structured Clinical Examination; ERAS, Electronic Residency Application Service.

### 3.2 Structure of the MSPE at Alfaisal

**Figure 2:**
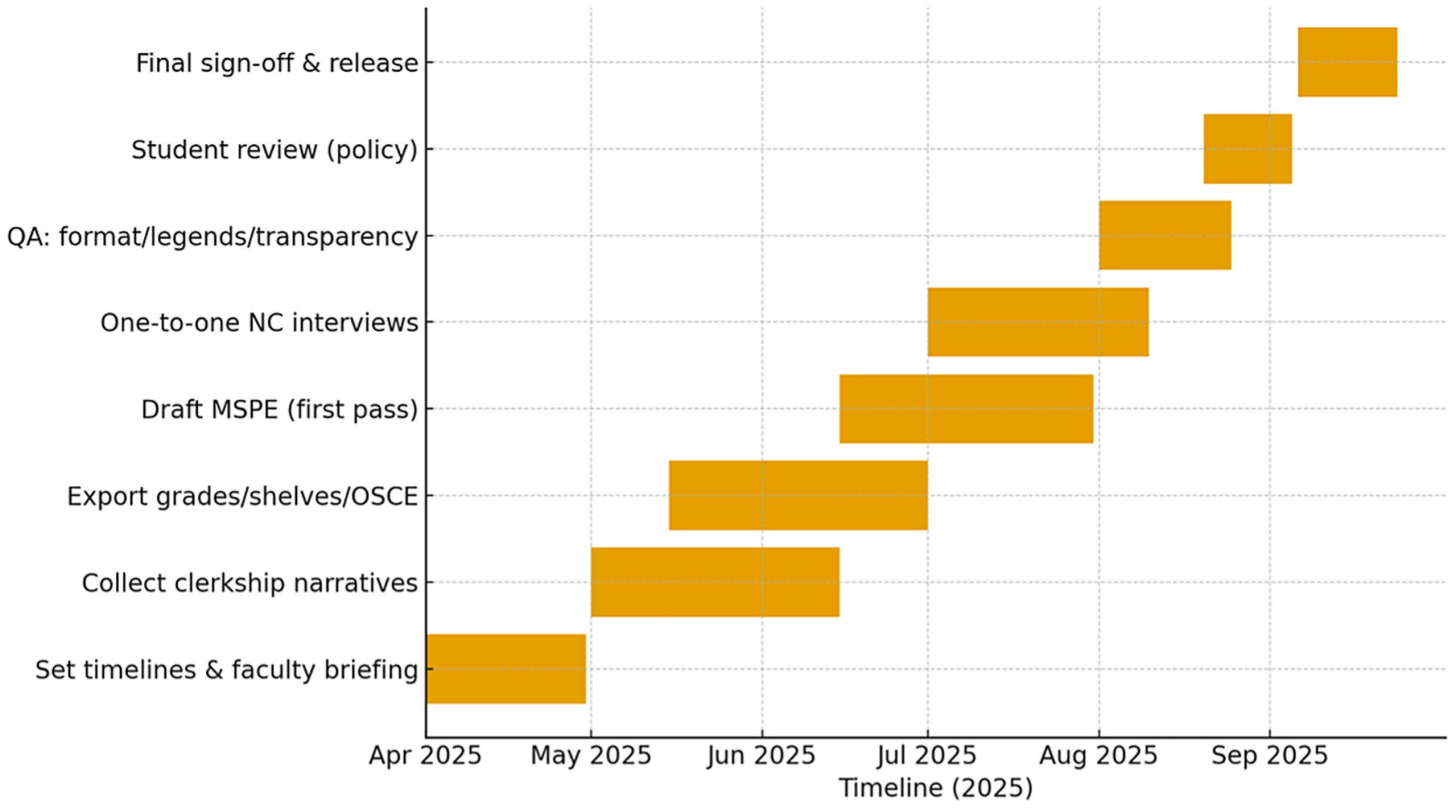
Process framework for Medical Student Performance Evaluation (MSPE) development and implementation at Alfaisal University. The framework illustrates the sequential and iterative stages involved in MSPE preparation, including institutional policy alignment, faculty development, standardized data collection from clerkships, narrative synthesis, multi-stakeholder review, and final release aligned with the ERAS timeline. The model emphasizes coordination between academic leadership, clinical departments, and Student Affairs to enhance transparency, consistency, and comparability in student performance reporting. Dates are illustrative and reflect alignment with a typical ERAS application cycle.

The MSPE is created through a multi-step process involving close coordination between the Office of Student Affairs, clerkship directors from all clinical departments, and faculty advisors. Every year, over 60 faculty and administrative staff members help prepare MSPEs, ensuring that content is cross-checked and uniform across departments. This level of faculty participation reflects a structured, multi-stakeholder approach consistent with recommendations in the MSPE literature (17). Individual student interviews are a unique feature of the Alfaisal process. Every graduating student meets one-on-one with the MSPE team leader to ensure that the Noteworthy Characteristics section accurately reflects their background, strengths, and objectives. The interview is not used to change evaluative content, but rather to verify accuracy in expressing the student’s background, context, and long-term goals. This technique helps to eliminate the discrepancies that often occur when students write their own NCs without institutional assistance, while also promoting a balanced, professionally written NC section. While Noteworthy Characteristics (NCs) are frequently regarded as one of the least standardized MSPE components nationally, Alfaisal’s individualized approach contributes to both accuracy and professionalism, aligning with recommendations encouraging institutions to develop NCs that are concise, meaningful, and consistent (13). The MSPE preparation process at Alfaisal, illustrated in Figure 2, spans from setting timelines and faculty development to final release, aligned with the ERAS 2026 cycle. Although developed within a single institution, this process framework may be adaptable to other medical schools seeking to implement or refine MSPE-aligned performance evaluations in non-North American contexts.

### 3.3 Data collection and integration of performance information

**Figure 3:**
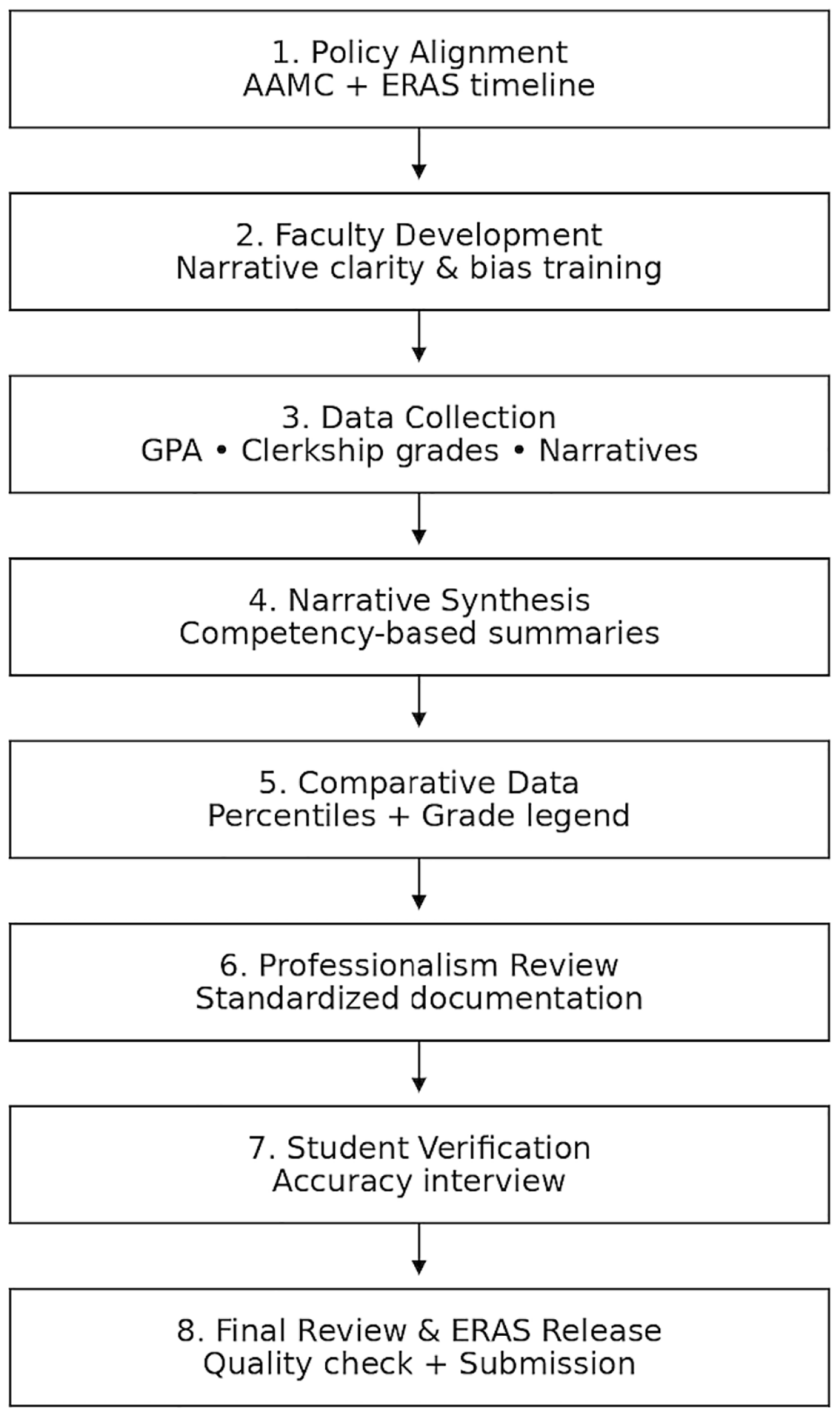
Operational workflow for Medical Student Performance Evaluation (MSPE) preparation at Alfaisal University. The diagram illustrates the structured and sequential process of MSPE development, including policy alignment, faculty development, standardized data collection, narrative synthesis, comparative data integration, professionalism review, student verification, and final ERAS release. The model highlights institutional safeguards implemented to enhance transparency and comparability.

Narrative comments from all clerkships are collected directly from clinical departments using a uniform internal format that corresponds to the AAMC guidance for including narrative assessments into the MSPE. Moreover, in recent years, the university has also attempted to regulate how clerkship narratives are written by providing faculty development sessions focused on clarity, eliminating imprecise adjectives, and reducing variability among departments. This is consistent with nationally acknowledged efforts to promote more structured and competency-linked narrative language. This step is critical because narrative variability has consistently been found as a limitation in MSPEs in many studies, restricting their value to residency programs (17). Alfaisal’s assessment approach consists of continuous clinical evaluation, OSCE performance, MCQ examinations, and organized feedback sessions. These components enable the MSPE team to provide a comprehensive picture of academic and clinical performance. Because unequal grading processes across institutions are a primary reason MSPEs differ in dependability (17), Alfaisal’s adoption of a well-defined grading system that is consistently applied throughout clerkships helps decrease interpretive uncertainty. The university also incorporates GPA and academic distinctions directly from the Assessment Office, which ensures that transcripts and MSPE content are consistent. USMLE scores are not automatically included in the Alfaisal MSPE. Their inclusion is conditional and based on institutional policy alignment with ERAS guidelines and explicit student authorization. The MSPE does not derive summary adjectives or comparative rankings from USMLE performance. Extracurricular activities, leadership roles, and substantial research accomplishments are sometimes included, but only when they contribute significantly to a student’s professional development. The operational workflow underlying this process is summarized in Figure 3. Data extraction is centrally coordinated by the Office of Student Affairs in collaboration with the Assessment Office to ensure concordance with official transcripts. Comparative performance categories (e.g., percentile or cohort position) are calculated using the full graduating class as the reference group, with grading legends explicitly defined. Narrative comments from clerkships are synthesized by the MSPE faculty lead to ensure clarity and consistency while preserving the original evaluative intent. Professionalism concerns are included only when formally reviewed through institutional academic or professionalism committees, with documentation of outcomes where applicable.

### 3.4 Adaptation for different applicant pathways

Although the MSPE’s overall framework stays consistent, slight changes are made based on a student’s selected specialty or region of application. These changes do not affect evaluative content but rather ensure that significant successes or clinical strengths are presented in a way that is appropriate for programs in the United States, Canada, or the region. Alfaisal’s student-specific interviewing process enables the MSPE to reflect each student’s career path while maintaining neutrality. This method is distinct from what numerous studies refer to as “one-size-fits-all” MSPE letters, which may fail to highlight specialty-relevant competencies (13).

### 3.5 Institutional outcomes and regional significance

Alfaisal University has achieved consistent residency match results in the area after putting in place a structured MSPE methodology. The MSPE’s organization and clarity are often mentioned in the literature as crucial for residency programs’ interpretability, even if a variety of criteria, such as student achievement and advising infrastructure, influence match performance. Program directors have repeatedly stressed the importance of well-structured MSPEs with transparent grading information, coherent narratives, and clear institutional context, as opposed to documents with inconsistent grading or ambiguous language, even though MSPE quality is only one part of the entire residency application. According to Brenner et al. (13), these components are highlighted in the Alfaisal MSPE framework and are frequently seen as improving the MSPE’s value during application evaluation.

Alfaisal’s experience offers a useful illustration of consistent MSPE implementation outside of North America in an international setting where formal advice is still scarce and institutions frequently rely on local modifications of U.S.-based templates. There are currently very few published accounts of MSPE practices in the Middle East, and there are especially few peer-reviewed publications describing MSPE implementation in GCC institutions. In this regard, the Alfaisal experience offers a thorough institutional illustration of how a framework that was developed abroad can be modified for a local educational setting. Such descriptions could help organizations looking to enhance the structure, transparency, and interpretability of their own performance evaluations as residency programs increasingly assess candidates from a variety of training systems.

## 4 Regional and global perspectives on the MSPE

A focused PubMed search combining Medical Student Performance Evaluation–related terms with country-specific MeSH headings for Saudi Arabia, Egypt, Jordan, Lebanon, Qatar, and the United Arab Emirates identified 40 publications. After screening these studies, it became clear that most of the literature in the region focuses on broader themes in medical education, such as the accuracy of student self-assessment, OSCE performance, simulation-based training, e-learning engagement, burnout, and curriculum development. Notably, none of the retrieved studies described the Medical Student Performance Evaluation (MSPE), the Dean’s Letter, or any comparable formal evaluation tool used in residency applications.

This absence of regional literature suggests that, although performance assessment is widely explored in Arab medical schools, the function of standardized summative evaluations, similar to the MSPE, has not yet been formally documented or studied. Understanding whether such evaluations are issued in Arab institutions, and how they are perceived by both local and international residency programs, represents an important gap for future research.

In contrast, performance evaluations similar to the MSPE are well established in various nations, despite significant differences in structure and level of standardization. In the United States, the MSPE is a required component of ERAS residency applications and follows a structured template created by the AAMC, with an emphasis on narrative synthesis and the inclusion of comparable performance statistics (22). In Canada, residency applicants use the Canadian Resident Matching Service (CaRMS) to submit a Medical Student Performance Record (MSPR) or Dean’s Letter; however, there is no standard national template, and the content and structure differ amongst medical schools (23). In the United Kingdom, applicants to the Foundation Programme are assessed using the Educational Performance Measure (EPM), a decile-based ranking of academic achievement that has historically been used alongside the Situational Judgement Test (SJT) to guide placement decisions (24). Shown in Table 3. To date, no peer-reviewed publications describe a nationally standardized MSPE-equivalent framework within Gulf Cooperation Council (GCC) countries or broader Middle Eastern medical education systems. While individual institutions may issue Dean’s letters or institutional summaries, there is no evidence of a region-wide standardized template comparable to the AAMC MSPE framework. The absence of published guidance or comparative studies underscores a significant gap in the regional medical education literature.

**Table 3:** International analogs to the MSPE (22–24).

Country/region	Instrument name	Mandatory?	Comparative data	Narrative standards	Residency use case	Reference notes
United States	MSPE (Medical Student Performance Evaluation)	Yes (ERAS/NRMP context)	Encouraged by AAMC guidelines	Structured by AAMC template	Central evaluation document in residency applications	MSPE defined as a comprehensive evaluation sent with ERAS applications; AAMC guidelines emphasize standardized sections and comparative data (22).
Canada	MSPR/Dean’s Letter (Medical Student Performance Record)	Yes (CaRMS context)	Varies by school; not standardized nationally	Varies; not uniform template	Principal document in Canadian residency applications through CaRMS	CaRMS describes an MSPR/Dean’s Letter as a required standing document but without a national template—format varies across schools. (CaRMS).
United Kingdom (UK)	Educational Performance Measure (EPM) & SJT	Yes (UKFP context; evolving)	Decile-based performance ranking	EPM includes deciles, degrees, publications	Used for allocation to Foundation Programme (historically alongside SJT)	The EPM is a ranked performance score based on deciles and additional achievements, used to allocate places in the UK Foundation Programme (24).
Arab region	Varies; early/*ad hoc* MSPE-like tools	No/varies widely	Limited; inconsistent regional standards	Not standardized; developing	Selective use in regional residency systems	No formal peer-reviewed regional standard; implementation varies and is not universally adopted; reflects how schools adapt MSPE frameworks locally.

This table compares the MSPE used in the United States with similar performance evaluation tools employed in other regions (Canada, UK, Arab world), outlining differences in implementation and data usage.

MSPR, Medical Student Performance Record; CaRMS, Canadian Resident Matching Service; EPM, Educational Performance Measure; SJT, Situational Judgement Test; GCC, Gulf Cooperation Council.

## 5 Challenges, insights, and future directions

### 5.1 Accuracy in reflecting student competencies

The Medical Student Performance Evaluation (MSPE) is supposed to be a comprehensive and objective summary of a student’s competencies throughout undergraduate medical education, potentially covering all core competencies (25). But a closer assessment reveals some major limitations. A main issue is the lack of standardization and transparency. Despite AAMC guidelines, there is huge variability in content, nomenclature, and grading systems used across institutions (15, 16). This includes 77 different key words (e.g., “Outstanding,” “Excellent”) with arbitrary and school-specific meanings. For example, “Outstanding” can mean students anywhere from the 32nd to the 99th percentile (7, 15). This makes inter-institutional comparisons impossible and undermines the document’s credibility as a true reflection of student competence (8, 16). Efforts to personalize the MSPE, like allowing students to write their own “Noteworthy Characteristics,” are seen by students as a way to highlight non-academic qualifications and hardships, but this is variably implemented across schools, which is another dimension of non-standardization (12).

Furthermore, narrative evaluations are often hindered by polite rhetoric, code words and systemic bias which disadvantages students (18). Studies show systematic differences in language used based on gender and race; for example, women are described with “compassion” words like “caring” more often while men are described with “standout” adjectives like “exceptional” (25). These differences persist even after controlling for USMLE Step 1 scores, indicating that bias rather than actual performance is driving these evaluations (25). Reporting of core competencies like professionalism is becoming more common but lacks universal definitions and narratives often omit constructive criticism or examples of unprofessional behavior, presenting an overwhelmingly positive but inaccurate picture (11, 26, 27). Thus, the MSPE’s predictive value for residency performance is limited, and program directors (PDs) do not trust the subjective evaluations especially for professional behaviors (6, 11, 20).

### 5.2 Correlation with standardized test scores

The MSPE’s limitations have made us rely more on standardized exams, especially USMLE Step 1. Historically, programs directors (PDs) have ranked the MSPE low in importance, sometimes 20th among selection factors, while giving much more weight to USMLE scores (20, 28). This is because the MSPE is inconsistent and lacks comparability (7, 20). When data is missing, PDs lean on exams, which they perceive as more objective (7, 17).

The relationship between MSPE content and exam performance is complicated. One study showed MSPE quintiles predict residency performance at a comparable level to Step 1, with each being an independent predictor (29), other evidence shows a major overlap. Some schools explicitly include USMLE scores in their MSPE adjectives or class rankings, so they amplify the effect rather than offer an independent view (27). With Step 1 now being reported as pass/fail, PDs are looking more to the MSPE for comparative data. But current issues such as grade inflation, vague descriptors, and non-standardized comparisons, undermine its credibility as a counterweight (15).

### 5.3 Shift toward data-driven and statistical approaches

There is growing evidence to move the MSPE towards a data driven model. PDs are asking for inclusion of objective, comparable metrics, such as quartiles, class rank and standardized NBME shelf exam percentiles (6, 18). Narrative sections can still add value if structured around competency frameworks like ACGME milestones, Entrustable Professional Activities (EPAs) and the RIME model which emphasize observable, measurable behaviors (6, 18).

New statistical and machine learning (ML) approaches are emerging but face challenges. Commercial ML tools tested on MSPE narratives failed to rank students reliably because of the “range restriction” of uniformly positive language (30). Future tools will need MSPE-specific training datasets that can detect subtle gradations. In the meantime, integrated approaches combining MSPE data (e.g., quintiles), USMLE scores and other markers like Alpha Omega Alpha (AOA) status in regression models show promise to predict resident performance better than any single metric (29).

### 5.4 Opportunities for standardization across institutions

There are significant opportunities to improve the MSPE by standardizing across all medical schools. The 2017 AAMC guidelines led to measurable progress by adoption of a 6-section format, usage of shorter documents, adding more comparative data and professionalism statements, and promoting narrative’s co-location with grading schemes (26, 27). For example, the percentage of MSPEs with dedicated professionalism sections jumped from 12% in 2015 to 76% in 2018 (26).

Nonetheless, full compliance and standardization remain elusive. Challenges persist, including inconsistent graphical data presentation, use of appendices contrary to guidelines (37.2% of MSPEs still use them despite AAMC recommendations to eliminate them) and no consensus on defining and reporting professionalism and remediation (26, 27). To correct this, future efforts must require strict adherence to AAMC guidelines, eliminate appendices, and standardize visual presentation of comparative data (27). In addition, attention must be paid to the narrative content itself. Faculty development is identified as a critical intervention; workshops using deliberate practice to rewrite biased narratives and apply competency-based frameworks have shown high efficacy and participant satisfaction and are a scalable model for national training (18). Finally, we must standardize key metrics by using school-specific percentiles for clerkship grades and a unified system for summary adjectives with clear legends to level the playing field and make it equitable for all students, especially those from smaller schools (15, 16, 27). In light of persistent variability, a pragmatic path forward may be the articulation of a minimum core dataset for MSPE-equivalent documents. Such a dataset would include a transparent academic history summary with explanation of grading schemas; clearly defined comparative performance data contextualized within the graduating cohort; structured clerkship narratives aligned with competency domains; an explicit professionalism statement, including documentation of remediation where applicable; disclosure of how clerkship grades are derived; and a concise, verified section highlighting noteworthy student characteristics. Establishing these foundational elements across institutions could improve interpretability and cross-institutional comparability while preserving necessary narrative flexibility.

## 6 Conclusion

Despite substantial improvements over the previous 20 years, the MSPE’s influence is still constrained by inconsistent adherence to suggested standards, inconsistent grading procedures, and ongoing difficulties with narrative assessment. A reliable, transparent, and comparable MSPE is more important than ever since residency programs are depending more and more on non-exam criteria in the age of pass/fail Step 1 reporting. Alfaisal University’s experience demonstrates how the MSPE framework can be applied outside of the US when institutions emphasize faculty development, structured data collection, and clarity. Future study in the Middle East has a significant opportunity due to the lack of regional literature on MSPE-equivalent documents. To ensure that the MSPE serves as an equitable and significant evaluation instrument for residency selection, more cooperation, more precise definitions of critical competences, and systematic standardization across institutions will be crucial.
